# Epidemiology of Achilles tendon surgery in Italy: a nationwide registry study, from 2001 through 2015

**DOI:** 10.1186/s12891-020-03688-2

**Published:** 2020-10-17

**Authors:** UG Longo, Giuseppe Salvatore, Laura Risi Ambrogioni, Eleonora Cella, Vincenzo Candela, Arianna Carnevale, Emiliano Schena, Massimo Ciccozzi, Nicola Maffulli, Vincenzo Denaro

**Affiliations:** 1 Department of Orthopaedic and Trauma Surgery, Campus Bio-Medico University, Via Alvaro del Portillo, 200, 00128 Trigoria, Rome, Italy; 2 Medical Statistics and Molecular Epidemiology, Campus Bio-Medico University, Via Alvaro del Portillo, 200, 00128 Trigoria, Rome, Italy; 3 Unit of Measurements and Biomedical Instrumentation, Campus Bio-Medico University of Rome, Rome, Italy; 4 Centre for Sports and Exercise Medicine, Barts and The London School of Medicine and Dentistry, Mile End Hospital, 275 Bancroft Road, London, E1 4DG England; 5 Department of Musculoskeletal Surgery, University of Salerno School of Medicine, Surgery and Dentistry, 84121 Salerno, Italy

**Keywords:** Achilles tendon, Rupture, Registry, Surgery, Epidemiology, Prevalence

## Abstract

**Background:**

This study aims (1) to estimate the yearly number of Achilles tendon (AT) surgeries in Italy from 2001 to 2015 based on official hospitalization records; (2) to investigate the eventual presence of geographical variation in equity in access to AT surgery between three macroregions of Italy (North, Center and South); (3) to perform statistical projections of the number of AT procedure volumes and rates based on these data.

**Methods:**

We analysed the National Hospital Discharge records (SDO) maintained at the Italian Ministry of Health for a 15-year period, from 2001 through 2015. These data are anonymous and include the patient’s age (evaluated in the class of age), sex, census region, the region of hospitalization, length of the hospitalization, public or private reimbursement and diagnosis.

**Results:**

During the 15-year study period, 118,652 AT repair were performed in Italy, whose peak of incidence was in 2010. More than half of AT repairs was performed in the North of Italy (52.1%), while 27.2% was performed in the South of Italy and 20.6% Center of Italy. The projection model predicted a slight growth of 2.65% in 2025 in comparison with 2015.

**Conclusion:**

The current study provides detailed information about the national population-weighted incidence of AT surgery, distribution and projection. The peak of average age was 35–45 year. The majority of AT procedures was performed in the North of Italy. The projection model predicts a slight growth of AT surgery by 2025. Furthermore, this 15-year nationwide registry study shows that the age of incidence of AT injuries shifted from 30 to 40 to 35–45 years compared to the available literature. The higher prevalence of AT surgery was found in men during the working age. Moreover, a low rate of procedures in pediatric and elder age classes was observed.

## Background

The Achilles tendon (AT) is the largest and strongest tendon in the human body. AT ruptures account for more than 40% of all tendon ruptures, and they are most common in males [1]. The management of this type of injury often demands surgical intervention [2–4].

AT ruptures can be distinguished into acute, when diagnosed or treated within 6 weeks, or chronic [2, 5]. However, since up to 25% of acute AT ruptures are misdiagnosed, they become chronic after four to 6 weeks and can not be handled as easily as acute ones [4, 6, 7]. The management of chronic AT ruptures is technically more challenging due to the greater gap between the two ends of the tendon and the degeneration of the contiguous soft tissues [2, 5]. This complexity can be experienced especially in females who seem to have a higher rate of complications, a longer recovery time and an increased risk of further surgery [7–10]. Consequently, the quality of health services and their efficiency in providing early diagnosis, treatment and follow-up clinical care would be crucial in avoiding delays in AT surgery [7–10].

It is known that spontaneous injuries are frequent and occur more often in athletes than those who lead a sedentary life (in a ratio of 3:1) [1, 2, 5]. However, in the last decade, some studies have reported a reversal of the trend with an increase in the number of injuries in sedentary individuals ageing 30 to 40 years, in those who play sport occasionally and in older adult engaged in high-demand sports [1, 11–15]. A possible hypothesis that could explain these variations in epidemiological trends is to be found in the poor quality and reliability of the data used. Indeed, countries without national registry data can not infer statistics based on their own results. However, the incidence of the disease is calculated from those of other regions, and this can lead to substantial variations in rates. Unfortunately, comprehensive nationwide studies on incidence rates of AT surgery have only been reported for Denmark from 1994 to 2013 [16], Sweden between 2001 and 2012 [14] and Finland from 1987 to 2011 [17].

The epidemiological trend in the number of accesses to surgery can be useful to evaluate the economic effects of the disease and to analyse the costs and economic benefits, as well as to monitor the history of the disease itself. Indeed, the considerable decrease in the number of surgical procedures observed in previous national studies can be explained by the absence of significant differences between surgical and non-surgical management of AT ruptures demonstrated by recent high-quality studies [14, 17]. If further nationwide registry study, statistical projections and forecasts for the future could confirm these results showing that excellent clinical outcomes can be achieved through conservative treatment alone, a radical change in the management of AT rupture management would be necessary [16, 17].

Being aware that epidemiological trends are essential as they can influence AT ruptures management protocols, a national study was conducted to focus on the economic and demographic impact of AT surgeries from 2001 to 2015 and statistical projections and forecasts until 2025. To achieve these purposes, this investigation was subdivided into three objectives. The first objective was to estimate the annual number of AT surgical procedures in Italy from 2001 to 2015 based on official hospitalization registers to provide valid and accurate epidemiological data and to compare trends with all available registry studies. The second objective was to investigate whether there are differences between patients performing the surgery in the same region of residence and patients receiving the surgery in other regions than their residence one to highlight the potential presence of a geographical variation in the number of accesses to AT surgery between three macro-regions of Italy (North, Central and South). The third objective was to make statistical projections of the number of volumes and rates of AT repair procedures based on these data until 2025 to contribute to defining the development and clinical history of this disease.

## Methods

Data from National Hospital Discharge records (SDO) maintained at the Italian Ministry of Health for AT repair (ICD-9-CM 8364) from 2001 to 2015 were collected to perform this investigation. The anonymous data included the patient’s age (evaluated in the class of age), sex, census region (region of residence), the region of hospitalization, length of the hospitalization, public or private reimbursement and diagnosis. It was calculated the annual number of AT repair in the whole Italian population, analyzing the incidence rates using the annual population size obtained from ISTAT (National Institute for Statistics).

For historical and geographical reasons, the Italian regions are divided into three macro-regions: North, Center and South. The North includes the regions of the North–West (Liguria, Lombardy, Piedmont, Aosta Valley) and those of the North–East (Emilia–Romagna, Friuli–Venezia Giulia, Trentino—South Tyrol, Veneto). The Center includes the regions of Lazio, Marche, Tuscany, Umbria. The South consists of the regions of Southern Italy (Abruzzo, Basilicata, Calabria, Campania, Molise, Apulia) and the islands (Sardinia and Sicily). It was performed a descriptive statistic to evaluate patients census region, the region of hospitalization and region where patients had surgery. Procedures performed on patients residing in the same region of hospitalization were defined as “regional surgeries”. Procedures performed on patients not living in the same region of hospitalization were defined as “extra-regional surgeries”.

Analyses of estimated costs were based on the costs ascribed to diagnosis-related groups (DRGs), according to Ministerial Decree (December 18, 2008). In Italy, reimbursement is the same for all the procedures under DRG, regardless of the diagnosis, the complexity of the procedure, or the patient’s health status at admission. Projection of the trend in the number of AT repair in the next 10 years (2016–2025) was performed using the “Forecast” function in Excel (Microsoft) software.

## Results

**Fig. 1:**
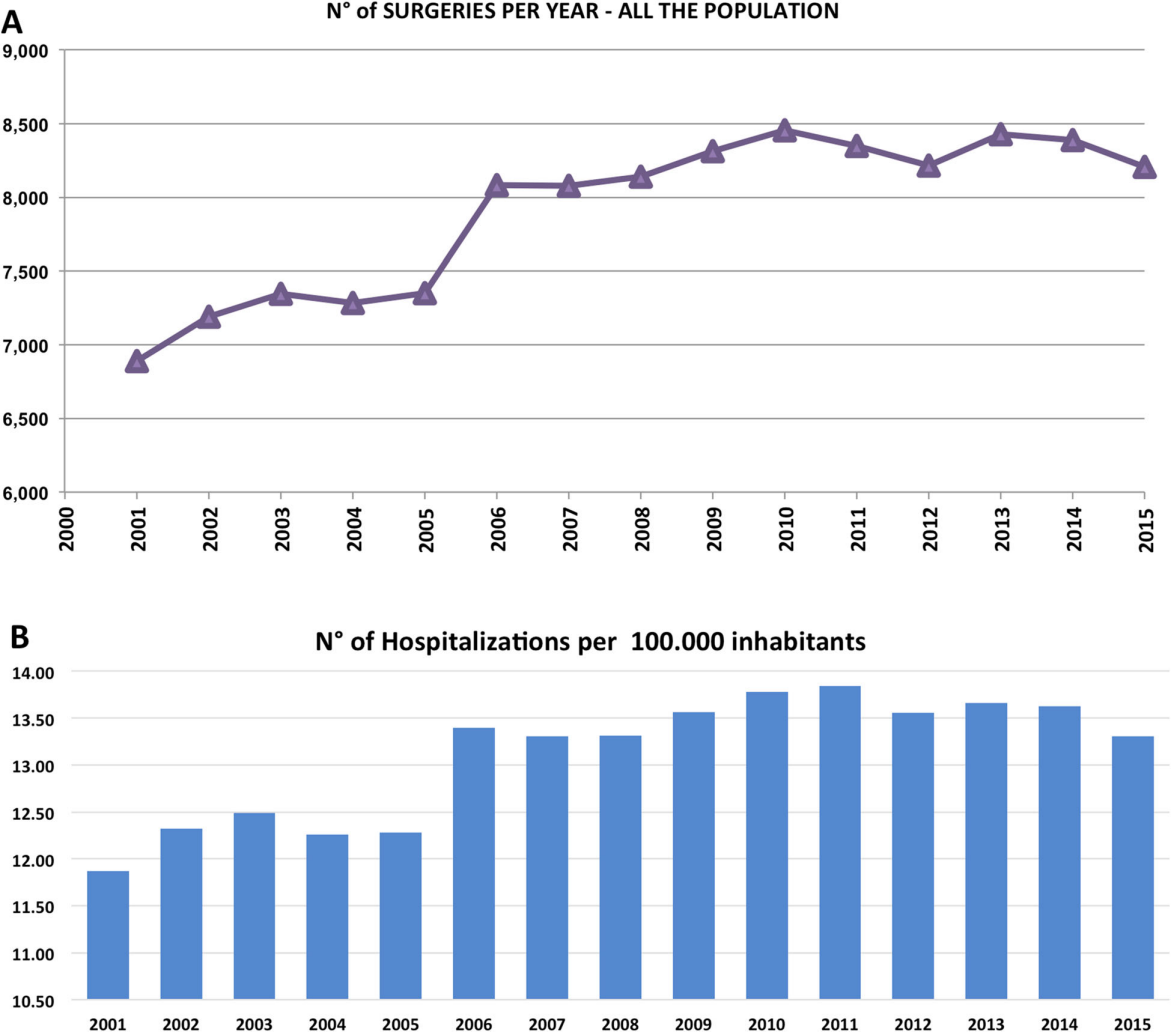
**a** Number of AT surgeries performed per year – all the population. **b** Number of hospitalizations per 100,000 inhabitants

**Fig. 2:**
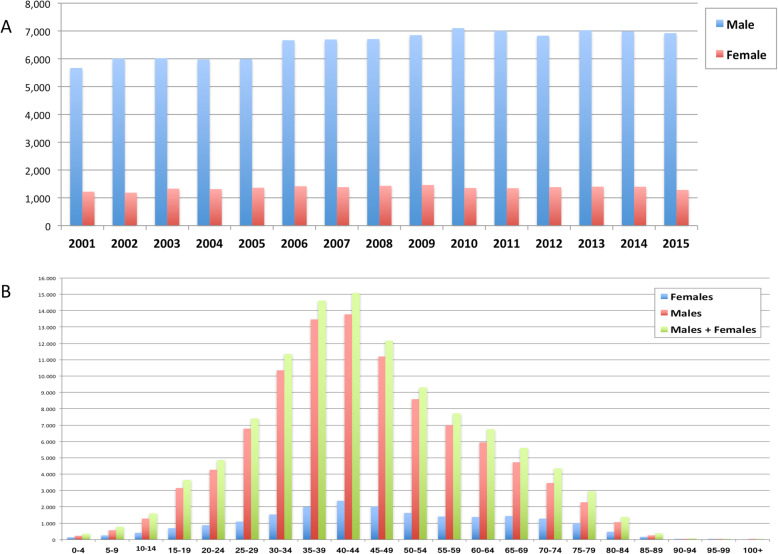
**a** Number of male/female patients who underwent AT surgery. **b** Distribution of male/female who underwent AT surgery for 15 years

**Fig. 3:**
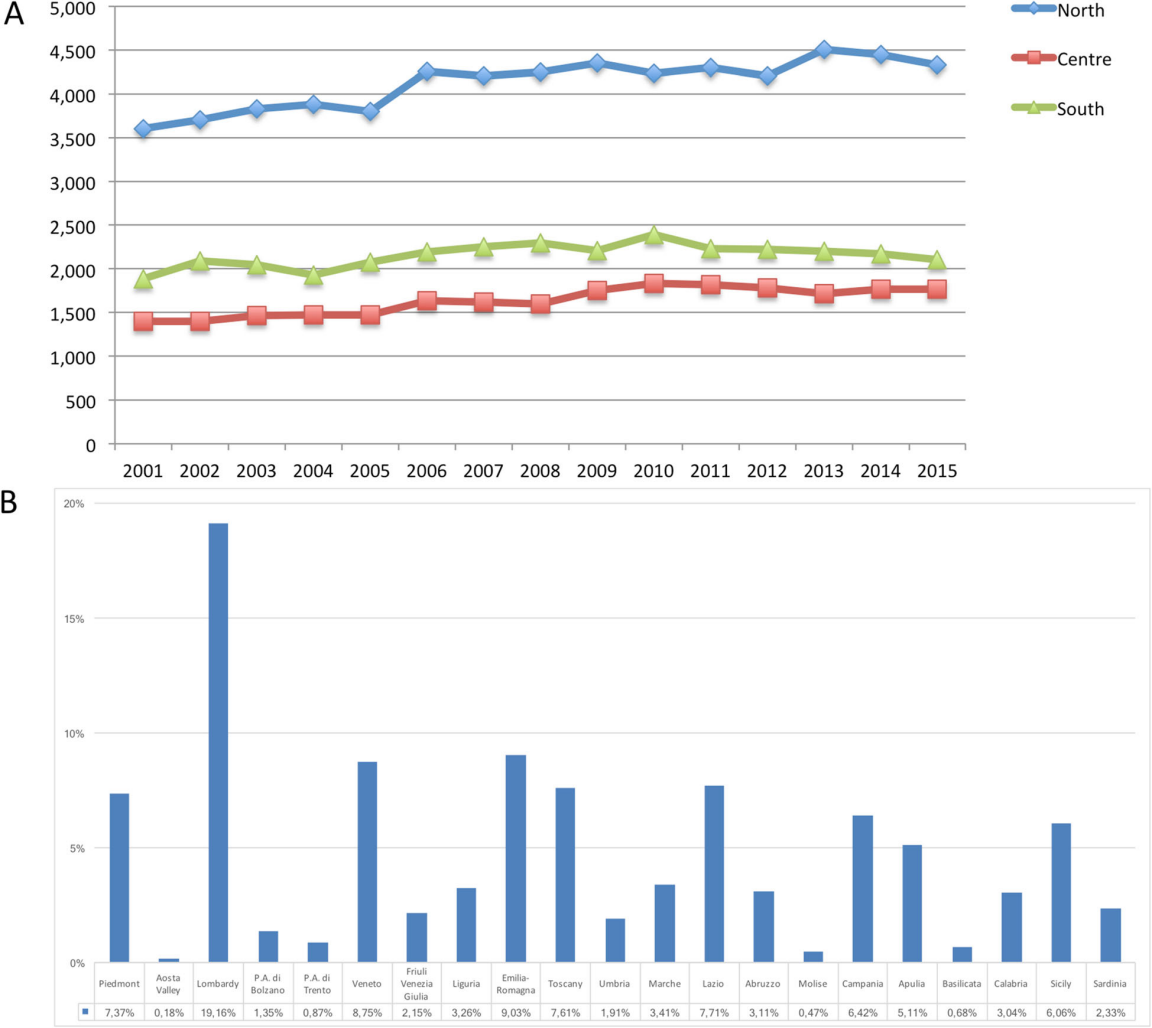
**a** Macroregional access to AT surgery. **b** Regional access to AT surgery

**Fig. 4:**
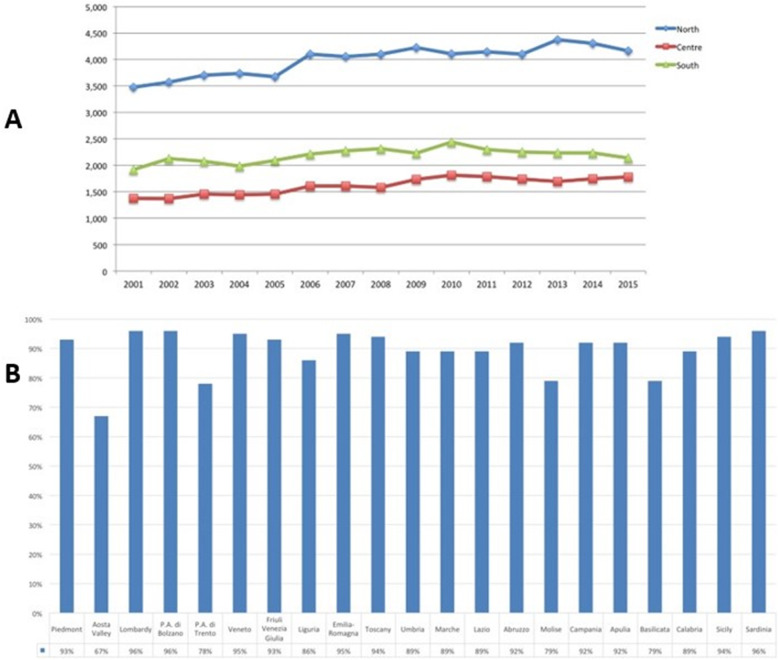
**a** Census region’s patients of procedures performed. **b** Detailed percentage of surgery performed in the region of residence

**Fig. 5:**
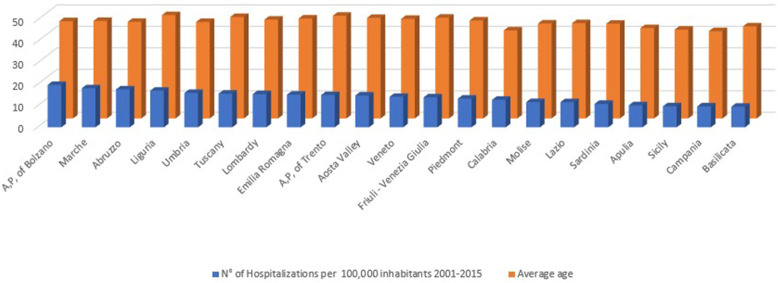
Influence of the average age of the regions on the incidence of AT surgery

**Fig. 6:**
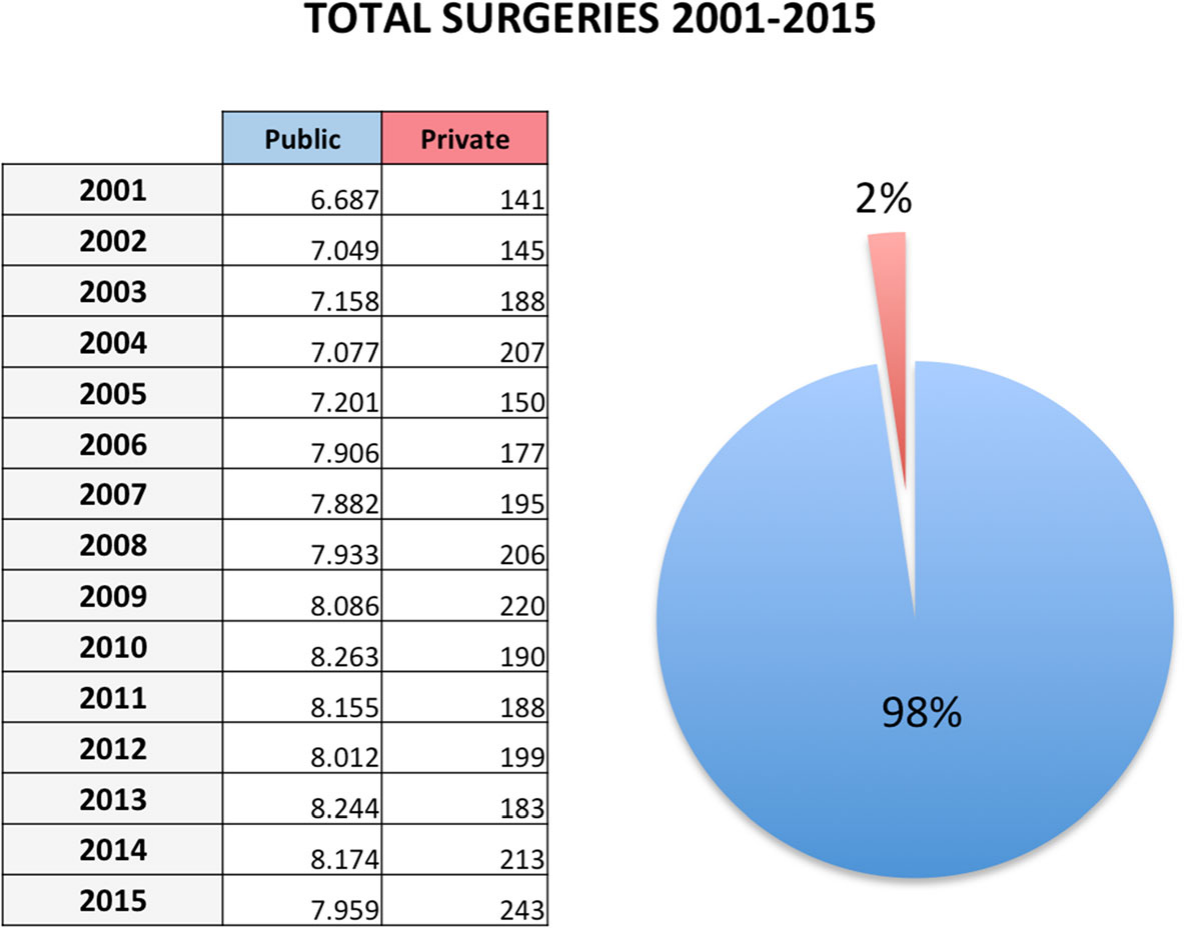
Public and private surgeries performed in the study-period

**Fig. 7:**
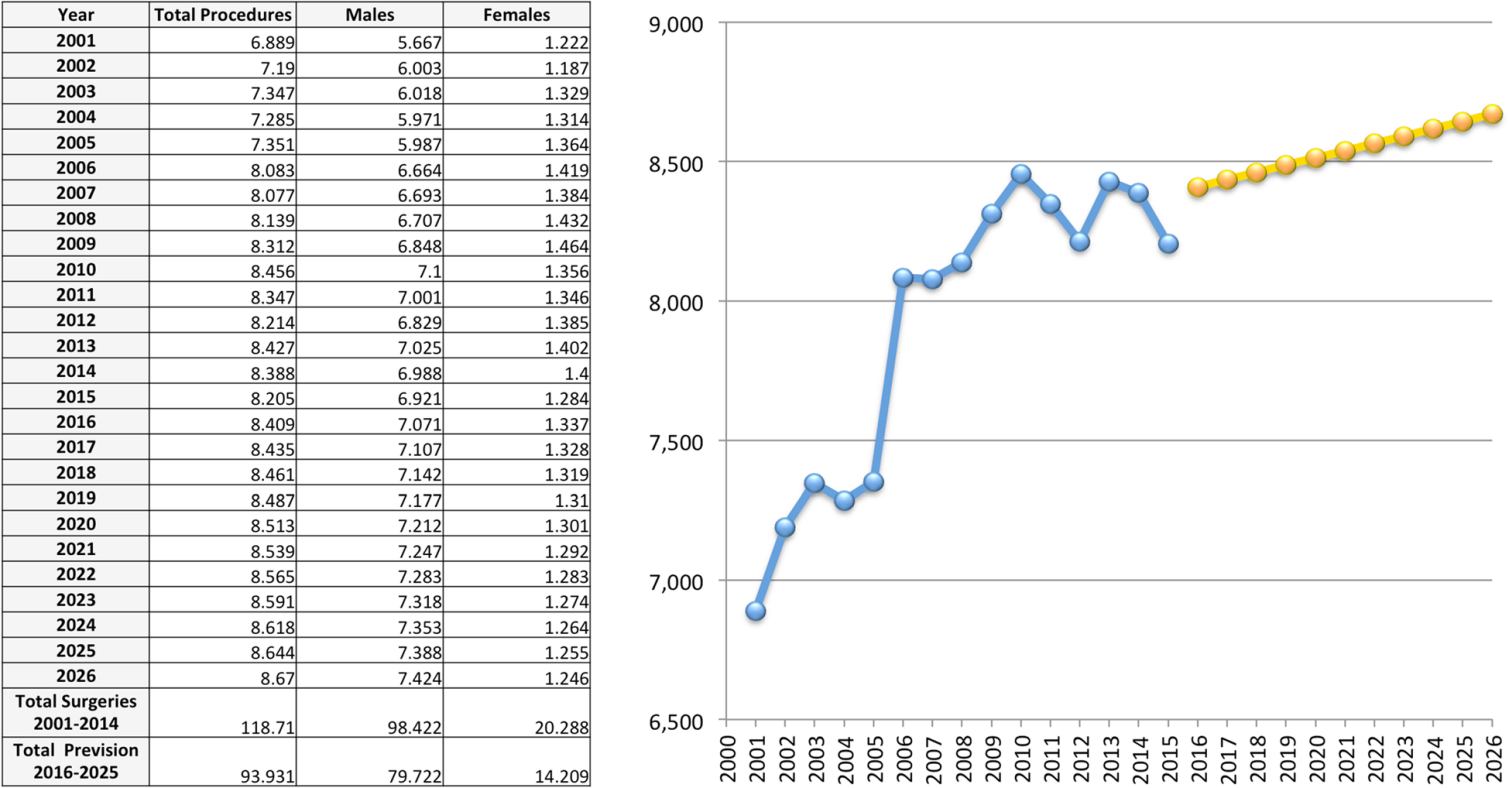
Trend’s prediction for 2015–2025

Overall, 118,652 AT repair were performed in the study period (2001–2015). The number of AT repair was less than 7000 in 2001 to more than 8000 in 2015, showing an increasing (Fig. 1 panel a and Table 1) with a peak in 2010. The cumulate period of incidence was 13.1 AT repairs for every 100,000 Italian inhabitants, observing the highest incidence in 2011 with 13.84 procedures for every 100,000 inhabitants (Table 1 and Fig. 1 panel b). The overall ratio of male/female was 4.85 (Table 1). The number of female patients undergoing surgical repair remained constant, while the number of male patients showed growth over the different years (Fig. 2, panel a). In Fig. 2, panel b the age classes involved in AT repair are shown. The age classes more represented are 35–39 and 40–44. AT repair procedure was very low in the pediatric and elder age classes. Most AT repairs were performed in the North of Italy (52.1%), 27.2% in the South of Italy and 20.6% in the Center of Italy (Table 1 and Fig. 3 panel a). There was a growth in each macro-region, but the more evident was in the North of Italy than the other macro-regions. The region with most procedures carried out was Lombardy with 19.16%, followed by Emilia-Romagna (9.03%) and Veneto (8.7%). All these regions are from the North of Italy. 51.2% of surgeries were carried out on patients living in the North, 20.7% on patients residing in the Center and 28.1% on patients living in the South (Table 1 and Fig. 4). 94% of patients living in the North underwent surgery in the macro-region of residence, 91% of patients living in the Centre underwent surgery in the macro-region of residence, and 92% of patients living in the South underwent surgery in the macro-region of residence. Figure 4 panel b shows in detail, the percentage of surgery performed in the region of residence. All the regions had a percentage greater than 78% except Aosta Valley with 67% but significantly high (see also Table 2). It is possible to define this surgery as a regional surgery. When data are adjusted for regional population density, the highest number of procedures during 2001–2015 is recorded in the A, P, of Bolzano (19.8/100000 inhabitants), in Marche (18.2/100000 inhabitants) and Abbruzzo (17.7/100000 inhabitants). All data concerning the number of hospitalizations per 100,000 inhabitants are summarized in Table 3 and shown in Fig. 5. Furthermore, it was observed that the increase in age positively influences the number of regional procedures performed (*r* = 0.67). In all regions, the average age was between the fourth and fifth decade of life, ranging from a minimum of 40.7 years in Campania to a maximum of 48.1 years in Liguria (Table 3, Fig. 5). The median length of hospitalization was 3 days in 2001, remained constant up to 2012 when there was a decreasing to 2 days (Table 1). In the study period, the observed overall ratio of public AT repair was 98% (Fig. 6). The predicted model projected a constantly increasing trend in the number of AT repairs in the next 10 years (2016–2025) (Fig. 7). According to this model, there will be a slight growth of 2.65% in 2025 compared to 2015. Analyzing the information on mechanisms of ruptures, the percentage of traumatic event leading to surgery showed a peak in 2001 (44.30%), with a decreasing trend to 40.66% in 2015 (Fig. 7).

**Table 1:** Demographics

	2001	2002	2003	2004	2005	2006	2007	2008	2009	2010	2011	2012	2013	2014	2015	TOTAL	
**Total Population**	56,993,742	57,321,070	57,888,245	58,462,375	58,751,711	59,131,287	59,619,290	59,942,270	60,340,328	60,516,381	59,394,207	59,685,227	60,782,668	60,795,612	60,665,551	890,289,964	
**N° of Hospitalizations**	6831	7190	7347	7285	7351	8083	8077	8139	8312	8456	8347	8214	8427	8388	8205	**118,652**	
**Male**	5621	6003	6018	5971	5987	6664	6693	6707	6848	7100	7001	6829	7025	6988	6921	**98,376**	
**Female**	1210	1186	1329	1314	1363	1418	1384	1432	1462	1356	1346	1385	1402	1400	1284	**20,271**	
**Male/Female Ratio**	4.65	5.06	4.53	4.54	4.39	4.70	4.84	4.68	4.68	5.24	5.20	4.93	5.01	4.99	5.39	**4,85**	
**Region of Hospitalization**																	
**North**	3567	3705	3830	3884	3800	4254	4203	4248	4353	4231	4301	4203	4503	4449	4329	**61,860**	*52.1%*
**Centre**	1391	1400	1467	1470	1472	1637	1623	1597	1752	1833	1818	1784	1719	1765	1768	**24,496**	*20.6%*
**South**	1873	2093	2050	1931	2079	2192	2251	2294	2207	2392	2228	2227	2205	2174	2108	**32,304**	*27.2%*
**Domicile of hospitalised Patients**																	
**From the North**	3445	3573	3703	3737	3670	4099	4052	4100	4222	4103	4142	4100	4374	4304	4160	**59,784**	*51.2%*
**From the Centre**	1366	1367	1453	1446	1455	1611	1606	1578	1734	1811	1784	1738	1695	1747	1776	**24,167**	*20.7%*
**From the South**	1900	2123	2073	1986	2091	2211	2276	2314	2230	2439	2296	2252	2234	2233	2138	**32,796**	*28.1%*
**N° of Hospitalizations per 100,000 inhabitants**	11.77	12.32	12.49	12.26	12.28	13.40	13.31	13.31	13.57	13.78	13.84	13.55	13.66	13.63	13.31	13.10	
**of the North**	13.46	13.85	14.18	14.11	13.75	15.26	14.93	14.96	15.30	14.78	15.23	14.97	15.74	15.48	14.99	14.73	
**of the Centre**	12.54	12.47	13.08	12.88	12.87	13.98	13.78	13.40	14.61	15.15	15.39	14.88	14.04	14.45	14.72	13.88	
**of the South**	9.26	10.33	10.03	9.57	10.07	10.65	10.93	11.09	10.68	11.66	11.14	10.92	10.68	10.68	10.26	10.53	
**Public Hospitalizations per 100,000 inhabitants**	11.733	12.297	12.365	12.105	12.257	13.370	13.221	13.234	13.401	13.654	13.730	13.424	13.563	13.445	13.119		
**Private Hospitalizations per 100.000 inhabitants**	0.247	0.253	0.325	0.354	0.255	0.299	0.327	0.344	0.365	0.314	0.317	0.333	0.301	0.350	0.401		
**Hospitalization lenght (days)**	3.0	3.0	3.0	3.0	3.0	3.0	3.0	3.0	3.0	3.0	3.0	2.0	2.0	2.0	2.0

**Table 2:** Regions

MIGRATION in 2001–2015 Timespan		Region of Hospitalization																					TOTAL for each Origin
		Piedmont	Aosta Valley	Lombardy	A.P. of Bolzano	A.P. of Trento	Veneto	Friuli - Venezia Giulia	Liguria	Emilia Romagna	Tuscany	Umbria	Marche	Lazio	Abruzzo	Molise	Campania	Apulia	Basilicata	Calabria	Sicily	Sardinia	
**Patient’s Origin**	**Piedmont**	8053	13	300	0	4	13	7	120	25	18	3	5	8	15	0	6	11	1	13	24	14	8653
	**Aosta Valley**	65	185	17	0	0	0	0	4	2	0	0	0	1	0	0	0	1	0	1	0	1	277
	**Lombardy**	123	5	21,017	10	25	176	13	54	248	63	3	32	28	18	2	28	35	10	23	28	41	21,982
	**A,P, of Bolzano**	2	0	6	1392	11	23	1	0	4	0	1	1	1	0	0	0	2	0	2	2	2	1450
	**A,P, of Trento**	0	0	77	37	894	109	2	0	11	5	0	2	2	0	0	1	1	0	0	0	2	1143
	**Veneto**	13	1	123	30	19	9607	101	6	127	22	2	11	11	8	0	6	10	2	6	7	7	10,119
	**Friuli - Venezia Giulia**	2	0	24	10	2	96	2362	1	12	2	0	3	6	1	0	1	3	0	1	6	2	2534
	**Liguria**	233	3	118	4	3	7	1	3480	23	142	0	1	3	1	0	1	1	0	4	0	3	4028
	**Emilia Romagna**	13	0	228	7	14	66	2	9	9145	34	3	46	7	10	2	6	9	1	8	7	11	9628
	**Tuscany**	23	0	58	5	3	7	1	83	166	7990	59	6	69	2	1	10	4	0	5	4	7	8503
	**Umbria**	2	0	6	2	2	1	3	4	27	128	1838	24	31	1	1	2	2	0	1	0	0	2075
	**Marche**	2	0	29	2	2	5	2	5	247	14	56	3676	26	46	2	3	4	0	3	1	3	4128
	**Lazio**	15	0	49	13	9	20	5	5	48	176	161	43	8452	308	21	56	19	5	26	18	19	9468
	**Abruzzo**	5	0	22	0	1	4	0	0	41	11	19	104	50	3146	8	9	6	0	0	1	2	3429
	**Molise**	1	0	3	0	0	0	0	0	10	1	3	5	21	57	444	8	5	0	0	1	0	559
	**Campania**	14	1	54	2	0	14	3	9	99	64	15	10	155	25	42	7297	19	41	21	10	4	7899
	**Apulia**	12	0	72	4	2	20	2	7	110	36	39	39	38	18	28	16	5740	36	8	3	1	6231
	**Basilicata**	4	0	7	0	0	3	0	0	21	9	9	2	18	3	0	21	74	670	3	1	0	845
	**Calabria**	17	0	46	0	0	7	3	3	56	37	11	5	55	4	0	22	48	40	3400	49	1	3804
	**Sicily**	21	1	105	4	1	23	0	11	109	59	5	4	35	1	0	8	4	1	12	6949	3	7356
	**Sardinia**	6	0	23	0	1	4	1	9	11	13	4	4	13	1	0	4	2	0	0	0	2594	2690
**TOTAL for each Region**		8626	209	22,384	1522	993	10,205	2509	3810	10,542	8824	2231	4023	9030	3665	551	7505	6000	807	3537	7111	2717	116,801

**Table 3:** Number of hospitalizations per 100,000 inhabitants and average age

Regions	N° of hospitalizations per 100,000 inhabitants																Average age (years)
	2001	2002	2003	2004	2005	2006	2007	2008	2009	2010	2011	2012	2013	2014	2015	2001–2015	
A,P, of Bolzano	16.4	16.4	16.3	19.4	17.5	23.2	21.3	22.0	20.4	20.9	18.3	19.6	21.0	19.8	23.3	19.8	45.3
A,P, of Trento	12.8	13.4	13.1	11.3	14.0	21.3	12.3	17.7	12.8	17.5	14.0	17.1	16.2	15.9	15.3	15.0	47.8
Abruzzo	15.4	20.0	18.9	14.6	17.7	19.2	17.0	15.9	16.8	20.2	20.0	18.9	17.8	17.8	14.6	17.7	45.0
Aosta Valley	12.5	16.8	19.1	16.5	10.6	12.9	10.4	15.9	15.0	16.6	13.4	15.0	14.9	14.0	18.7	14.8	46.8
Apulia	9.7	10.1	9.4	8.8	10.5	10.8	10.5	10.9	10.0	11.4	10.6	10.4	10.7	9.7	10.4	10.3	42.1
Basilicata	9.2	11.6	8.6	7.4	6.7	7.5	10.9	11.8	7.9	10.0	9.0	9.2	10.4	12.1	12.1	9.6	42.9
Calabria	14.9	16.0	14.0	11.7	12.5	13.5	13.7	13.5	13.2	14.5	10.9	11.8	9.7	11.0	11.2	12.8	41.0
Campania	6.6	7.9	8.4	8.1	7.7	9.3	10.4	10.1	9.9	9.9	103.4	9.8	9.6	10.2	8.9	9.7	40.7
Emilia Romagna	14.2	14.8	14.4	14.5	14.4	15.7	15.4	15.4	17.0	16.0	15.8	15.2	15.9	14.8	14.5	15.2	46.5
Friuli - Venezia Giulia	14.3	13.4	13.5	15.6	12.3	14.2	14.3	15.0	14.8	12.9	14.6	14.5	14.6	13.3	12.3	14.0	46.9
Lazio	10.0	9.4	10.9	11.2	11.3	12.6	12.1	12.1	12.5	12.9	12.2	13.0	12.3	11.3	11.8	11.7	44.4
Liguria	14.7	14.8	15.2	15.0	12.3	16.2	17.9	16.7	17.8	18.5	16.1	17.7	21.5	21.2	20.1	17.1	48.1
Lombardy	13.7	14.2	14.9	15.4	14.7	15.9	16.7	16.5	15.8	14.7	16.4	15.3	16.2	16.2	15.5	15.5	46.0
Marche	15.6	19.0	19.6	17.6	17.2	18.2	19.6	18.2	18.8	18.6	18.9	18.0	17.0	19.6	17.2	18.2	45.4
Molise	5.3	9.7	12.8	10.6	4.1	11.9	14.5	13.9	12.3	14.9	10.2	12.8	18.2	12.7	12.8	11.8	44.2
Piedmont	10.8	11.4	12.4	11.2	11.9	14.7	12.6	13.1	15.0	14.4	14.7	15.2	15.6	13.9	13.3	13.4	45.6
Sardinia	9.0	8.5	9.3	9.3	9.4	9.9	10.1	12.1	11.2	12.5	12.7	12.8	12.7	11.9	12.6	10.9	44.1
Sicily	8.9	9.1	9.1	10.4	10.9	9.8	9.9	10.1	10.2	11.0	10.1	9.7	9.9	9.3	9.0	9.8	41.3
Tuscany	15.0	14.2	13.7	13.1	13.2	14.6	14.9	13.9	17.1	18.2	18.2	16.4	17.3	17.4	17.6	15.7	47.2
Umbria	13.6	13.9	14.7	17.4	16.3	18.2	16.4	17.2	16.9	18.0	17.9	16.4	12.5	14.2	16.9	16.0	44.9
Veneto	14.6	14.8	15.1	14.5	15.0	14.5	14.1	13.7	14.2	14.6	12.8	13.0	13.9	14.4	13.8	14.2	46.4

## Discussion

This 15-year nationwide registry study is the first comprehensive, national investigation of AT surgery ever done in Italy. All information on dates, diagnoses and number of AT surgeries performed during the study period was obtained from the national register, which is compiled through a mandatory reporting system for the entire population.

Italy has 60.3 million citizens [18], who resides in twenty regions and two autonomous provinces (Trento and Bolzano) [19]. The population-weighted incidence rate for AT surgery was 13.1 AT repairs per 100,000 Italian inhabitants. Based on the population density for each region, the highest incidence rates during the entire study period were observed in the Autonomous Province of Bolzano, Marche, and Liguria. The peak incidence was observed in 2011 with 13.84 interventions per 100,000 inhabitants, suggesting both an increase in the number of interventions performed during the study period (2001–2015) and the relevance of AT tears as a public health problem. The present study has highlighted that the annual rate of surgery continues to grow as the average age of the Italian population rises. Despite the stable increase in AT surgical procedures, the trend has slowed considerably over the last 5 years, suggesting that non-surgical management may be a feasible option [20]. However, it is difficult to compare our results with those of the literature, as we are aware of only three other nationwide registry studies available on AT surgery [14, 16, 17]. 2011 was the year of the peak of incidence of AT surgery in both Italy and Finland with 13.1/100,000 and 20.5/100,000 person-years, respectively [17]. The Danish registry study recording 33,160 AT ruptures reported a statistically significant decrease in surgical treatment for younger men and women [16]. The study on the Swedish National Register was conducted from 2001 to 2012 and, in accordance with the two previous studies, showed an increase in the percentage of surgical interventions (from 33 to 77%) [14]. The following years showed a marked downward trend in the rates of AT surgery, suggesting a potential decrease in the incidence of surgical treatment [16, 17]. The Italian incidence AT surgery rate that has slowed considerably during the past 5 years has confirmed this expected decline.

The results of the present investigations are in agreement with the literature and confirmed the higher prevalence of AT surgeries in men (overall ratio of male/female was 4.85) and a lower rate of AT repairs in pediatric and elder age classes (Fig. 2 panel B) [1]. Findings from the present investigation have shown a shift of the time of incidence from the third and the fourth decade, as described in the literature [11, 12], to the range of age of 35–45 years, confirming that AT repairs mainly performed in the work population. In an attempt to optimise health-care resources and reduce costs, a review of procedures during hospitalization days in recent years has highlighted the possibility of reducing the median duration of hospitalization from three to 2 days.

One of the founding principles of the Italian National Health Service (NHS), created in 1978, is the free access to health care for its population. However, to achieve this objective, it is necessary to overcome socioeconomic barriers at the local, regional and national levels combining public financing with a mixture of public and private supplies [19, 21]. Italian NHS budget is transferred to the regions that manage 79% of public hospitals. Health-care providers, both public and private, (i.e. a hospital, outpatient surgery clinic) are rewarded through a free system based on a classification of procedures at fixed tariffs established by the regions or the Ministry of Health [19]. Moreover, reimbursement from other regional funds is only provided for public hospitals, and this can lead to cross-border mobility and facilitate extra-regional procedures. Indeed, at the end of the year, health-care providers that performed extra-regional surgeries have an extra budget that is outside the fixed tariff that could lead to a quasi-market health care system in continuous research of additional source of revenues [22]. Since 1994 in Italy DRGs Hospital Tariff System has been applied and it provides most devices that are fully covered by Italian NHS. Devices that are not included in this tariff system because they are too innovative or expensive are reimbursed separately, according to a system similar to regional and extra-regional procedures explained above [19]. This could be a triggering factor that can influence the flow of the patient from more impoverished regions (South) to more productive regions (North) and, therefore, lead to inequalities [22–25].

In fact, from the results of this national registry study, the geographical variations between three Italian macro-regions in rates of AT surgery have shown that most of all AT interventions were performed in the North, especially in Lombardy (19.16%) and that Italian patient preferred this type of surgery in the same region/macro-region of residence. The Italian health-care model follows the Beveridge model where private care activities are flanked but do not replace public care activities guaranteed by the State. However, it is well known that in the Northern macro-region, private care activities are more present, justifying the higher number of procedures performed in this territory [19, 21]. This hypothesis could also explain the high incidence rate of AT surgery performed in America, where the national health model is private [26]. Although the best management of AT ruptures is still under discussion, surgery is widely performed [26]. While it is recognized that operative treatment can reduce the risk of re-rupture compared to conservative treatment, doubts remain as to whether private care may have an incentive to perform operative treatment even in the absence of conclusive evidence [26]. Despite that, the present investigation sustains that equity in access to health-care in the Italian NHS is more efficient than private providers because the total amount of private procedures remained about 2% over the 15-year study period. According to the current NHS reimbursement policy, that could be explained with the unnecessary use of particular devices in AT procedures or to high efficiency of local hospitals. However, these findings can not explain why there is this high rates of AT procedures in Lombardy [19, 23]. The overview of spatial access highlights no differences in length of stay at the hospital, public or private reimbursement and region of hospitalization [27]. Therefore, based on these findings, it is possible to classify AT procedure as a regional surgery.

Even though this nationwide registry study is the first comprehensive, national investigation of AT surgery ever done in Italy, some potential limitations should be considered. Our results could be affected by possible changes in coding practices, but we are unaware of whether they would have significantly impacted our findings. However, the same codes are currently being used by governmental and nongovernmental public reports. The geographical variations among three Italian macroregions in AT repair rates may suggest inefficiency of the collection of unmet needs or over-indication in areas with different AT surgery rate. This could depend on patients or surgeons but, in both cases, potential inaccuracies in the coding of the diagnoses or procedures can not be clarified from the database analysis. The interpretation of our results should consider that Italian registry does not record non-surgical procedures and, therefore, we cannot compare these data each other, even though the interest in surgical and non-surgical management of AT ruptures is increased. Furthermore, codes used during our 15-year period study did not allow differentiation between surgical procedures, such as minimally invasive or open surgery [28], that compromise the possibility to cross data from different AT injuries.

This study would not have been possible without the availability of a reliable and worthwhile population-based registry and, therefore, we would remark to the surgeon community the importance of detailed clinical documentation. Through accurate data, it is possible to improve the accuracy and clinical relevance of administrative codes related to AT surgery and, in the same way, to all the procedures [29].

## Conclusions

This study represents the first detailed epidemiological investigation of AT surgery ever done across the entire Italian population able to confirm the socioeconomic burden of AT surgery that affects the male population, especially those ageing from 35 to 45 years. There is evidence of equity in access to AT surgeries across macroregions of Italy, as Italian patients could receive this type of surgery in the same region/macro-region of residence. However, it remains unexplained why the incidence of this surgery was higher in the North of Italy. According to the prediction model, AT surgery will have a slight growth in 2025 than in 2015. The current study, therefore, provides detailed information about the national population-weighted incidence of AT surgery, distribution, and projection. Our findings can now be used as an update of the clinical evidence base for Italian health-care planning improving awareness, health-services infrastructure, and accessibility.

## Data Availability

The data that support the findings of this study are available from *Direzione Generale della Programmazione Sanitaria – Banca Dati SDO,* but restrictions apply to the availability of these data, which were used under license for the current study, and so are not publicly available. Data are however available from the authors upon reasonable request and with permission of *Direzione Generale della Programmazione Sanitaria – Banca Dati SDO*.

## Abbreviations

AT: Achilles tendon
SDO: National hospital discharge records
NHS: National health service
DRG: Diagnosis-related group
