# Correction to: Epidemiology of Achilles tendon surgery in Italy: a nationwide registry study, from 2001 through 2015

**DOI:** 10.1186/s12891-020-03746-9

**Published:** 2020-11-10

**Authors:** Umile Giuseppe Longo, Giuseppe Salvatore, Laura Risi Ambrogioni, Eleonora Cella, Vincenzo Candela, Arianna Carnevale, Emiliano Schena, Massimo Ciccozzi, Nicola Maffulli, Vincenzo Denaro

**Affiliations:** 1 Department of Orthopaedic and Trauma Surgery, Campus Bio-Medico University, Via Alvaro del Portillo, 200, Trigoria, 00128 Rome, Italy; 2 Medical Statistics and Molecular Epidemiology, Campus Bio-Medico University, Via Alvaro del Portillo, 200, Trigoria, 00128 Rome, Italy; 3 Unit of Measurements and Biomedical Instrumentation, Campus Bio-Medico University of Rome, Rome, Italy; 4 Centre for Sports and Exercise Medicine, Barts and The London School of Medicine and Dentistry, Mile End Hospital, 275 Bancroft Road, London, E1 4DG England; 5 Department of Musculoskeletal Surgery, University of Salerno School of Medicine, Surgery and Dentistry, 84121 Salerno, Italy

Correction to: *BMC Musculoskelet Disord* **21,** 687 (2020)

https://doi.org/10.1186/s12891-020-03688-2

Following publication of the original article [1], the authors noticed that the author Umile Giuseppe Longo’s name was incorrectly modified to “UG Longo” in the final proof. The original article [1] has been updated.
